# Beyond deadlines and deliverables: Identifying barriers and facilitators to enhance the PROMICE of translational teams

**DOI:** 10.1017/cts.2025.64

**Published:** 2025-04-11

**Authors:** Whitney A. Sweeney, Maria Hernandez, Elizabeth S. Burnside, Josie Hintzke, Kayla Lemmon, Allan R. Brasier

**Affiliations:** Institute for Clinical and Translational Research, School of Medicine and Public Health, University of Wisconsin-Madison, Madison, WI, USA

**Keywords:** Project management, translational teams, center of excellence, education and training, career development

## Abstract

**Background/Objective:**

The Clinical and Translational Science Awards (CTSA) Program supports a national network of medical research institutions working to expedite the development of treatments and interventions. High-performing translational teams (TTs) involving inter-institutional collaborations are critical for advancing these evidence-based approaches. However, management of these complex teams can be difficult, and tailored project management may help TTs overcome the unique challenges they face.

**Methods:**

We conducted qualitative interviews with 14 dedicated project managers (PMs) from six CTSAs to learn more about their experiences with TTs. Information derived from the thematic analysis of the data was used to identify barriers and facilitators for effective project management.

**Results:**

Barriers included a lack of institutional support, communication issues, pushback, role confusion, and a need for agility. Facilitators included transparent communication, supportive team environments, shared leadership with autonomy, and opportunities for professional development. The PMs interviewed for this study provided descriptions of their work that depicted a more expansive view of project management than the more traditional approach focused on meeting deadlines and managing deliverables.

**Conclusion:**

Our findings have been used to inform development, training, and guidance for an innovative project management resource, the Project Management Innovation Center of Excellence (PROMICE) recently launched at the UW-Madison Institute for Clinical and Translational Research (ICTR). Through the development of a dedicated career path, PROMICE recognizes the value that PMs bring to translational science and provides the support that they need to be innovative, leading their teams to success.

The Clinical and Translational Science Awards (CTSA) program supports a national network of medical research institutions dedicated to developing, demonstrating, and disseminating innovations that enhance the efficiency and effectiveness of clinical translation [1]. High-performing translational teams (TTs) are critical for advancing evidence-based approaches that improve human health, but they often face unique challenges. TTs are typically interdisciplinary and composed of researchers, clinicians, trainees, and community partners. They function as a blend of academic knowledge generators and industry-like product developers, making their work complex and evolving as it progresses through the translational pipeline [2,3]. Large, multicomponent, and interinstitutional TTs face additional issues related to coordination and management. Because “…poor project management may be more to blame for the difficulties that arise in health-related research projects than flaws in research methodology,”[4] tailored project management may help TTs overcome the unique challenges they face.

Project management is traditionally described as the application of relevant knowledge, skills, and tools in a strategic manner to ensure that projects meet deadlines and provide deliverables while staying within budget [5,6]. There is much variability in how project management is implemented for TTs across CTSAs [6–8]. Often the Principal Investigator (PI), graduate students, postdocs, or early-stage faculty must assume these responsibilities. Unfortunately, training for the specific project management skills needed to effectively manage TTs is still lacking [9]. Some models propose a life-cycle continuum starting with graduate trainees and culminating in mature PIs, providing essential skill development appropriate for each career stage [7]. For any of these team members, it is difficult to accomplish their role in innovative research when simultaneously managing the cognitive burden of project management [10]. Engaging a dedicated project manager (PM), whose primary responsibility is to manage the project(s) as opposed to conduct the research, is one way to alleviate this burden.

Dedicated project management is often a part of successful innovation programs that help TTs move their scientific discoveries into clinical practice more efficiently [4,11]. Even 10% of a full-time PM can help teams develop a Collaboration Plan or project charter and generate quarterly reports [12,13]. They can also remove barriers that delay the translational process by acting as liaisons among all project partners including leadership and funding agencies. “Project managers bring rigor to the planning, organization and progress toward milestones.”[11] Moreover, PMs can play important roles in data management and sharing plans to enhance rigor and reproducibility. Though still not widely accepted in academia and often minimized, project management has the potential to serve as a “linchpin” supporting team science and accelerating translation and application [11,14].

Rarely, CTSAs provide dedicated project management resources in the form of Project Management Offices (PMOs), in contrast to Health Systems in which PMOs are common [6]. Interdisciplinary work “requires intentional alignment of incentives, management and resources to be successful” [11] and PMOs have been specifically designed to standardize project-related protocols and facilitate the sharing of resources, tools, and techniques [5]. There is evidence that PMOs can provide “a non-partisan, neutral coordinating mechanism” that helps organizations successfully complete major projects. Further, by facilitating communication and coordination among researchers, practitioners, and other broadly engaged partners, PMOs can also promote the diffusion of innovation throughout the translational ecosphere ensuring that innovative ideas are shared and implemented effectively [15].

Despite the obvious benefits of effective project management, many TTs are hesitant to fully embrace PMs in a dedicated role. Some teams see them as interlopers, there to oversee and micromanage. Some PIs feel that too great a focus on tracking and evaluation distracts from the research goals and others feel that “applying business practices to the management of science is still viewed as anathema.” [16]. The scientific method is iterative by nature, embracing intelligent failures and changing the research focus [17]. Some scientists feel that strict adherence to rigid project management standards common in industry stymies the creativity and fluidity of the scientific process. Thus, PMs for TTs need to be agile, prepared to deal with modified deadlines and deliverables or pivot to changes in the line of inquiry altogether [16].

It is also problematic that the dedicated role of a PM on a TT remains undefined, unlike other personas within the translational workforce. Gonzales *et al.* developed evidence-based profiles of roles (i.e., “personas”) to inform the use of educational resources and communication initiatives [18,19]. While elements of project management are found within some of the personas (e.g., Clinical Research Coordinator), there is no unique profile dedicated to a PM. This suggests a lack of consensus about how best to support those filling this vital role. Given the variability in how project management is implemented in TTs and the lack of role clarity, this study was designed to learn more about the experiences of dedicated PMs in TTs.

To effectively implement tailored project management that can accommodate the unique challenges faced by TTs, it is imperative to understand the key determinants that either facilitate or act as a barrier [20]. To learn more about the experiences of dedicated PMs on TTs, we conducted semistructured interviews of dedicated PMs from six different CTSAs across the national consortium (Table 1). The PMs interviewed worked with a variety of TTs during different developmental phases: team formation, knowledge generation, and translation [2]. In other words, they described working with nascent pilot awardees as well as more advanced product development teams. Information derived from the thematic analysis of the data was used to identify barriers and facilitators for effective project management, inform training, and provide guidance for an innovative project management resource at our CTSA. This article summarizes the study results and suggests future steps to better support the role of PMs on TTs advancing more innovative health solutions.

**Table 1.** Characteristics of study participants (*N* = 14)

Gender	
Female	11
Male	3
Clinical and translational science award region	
Northeast	2
Southeast	3
Midwest	8
Northwest	1
Education*	
BA/BS	2
MA, MS, MBA, or MPH	6
PhD	5
MD	1
Part of project management office	
Yes	6
No	8
Formal project management training**	
PMI PMP	7
Other formal training	9
No formal training	1

* BA = Bachelor of Arts, BS = Bachelor of Science, MA = Master of Arts, MS = Master of Science, MBA = Master of Business Administration, MPH = Master of Public Health, PhD=Doctoral Degree, and MD = Medical Degree.

** PMI = Project Management Institute, PMP = Project Management Professional Certificate.

*Note*: Three individuals had both formal certification from the Project Management Institute and additional formal project management training.

## Materials and methods

This study employed a mono-method qualitative research design using semistructured interviews. Fourteen dedicated PMs were selected using purposive sampling. CTSA administrators were contacted via a shared email mailing list and asked to provide information about how project management was handled at their institution. After communication by email or Zoom meeting, willing administrators provided the names of interested dedicated PMs at their home institutions. Additional participants were identified via snowball sampling with participating interviewees sharing names of other interested participants (see Table 1).

Each participant was interviewed for one hour by the lead investigator (WS). After describing how they were selected for their current position, participants were asked about the challenges they faced in meeting the needs of the TTs they worked with and to report on the support and resources their CTSAs provided. They were then asked to describe examples of notable successes. Finally, participants described the ideal project management environment based on their experience (see Table 2). This project was reviewed by the IRB and deemed exempt (ID#2023-1483).

**Table 2.** Questions asked during the 1-hour qualitative interview sessions

How were you selected for this position? What are the greatest challenges you see in terms of project management for the translational teams you work with or serve? What are the greatest needs? Please describe a time when your work as a project manager on a translational team was successful. What contributed to that success? How does your Clinical and Translational Science Award program support your translational teams in terms of project management? What are your aspirations? What would the ideal setup look like?

All interviews were transcribed verbatim, and participants were given pseudonyms to protect confidentiality. Data analysis involved coding transcripts for emerging themes. The project lead (WS) and research assistant (MH) coded all transcripts. Coding was conducted in three rounds. The first round used codes based on interview questions. During the second round, the answers to these questions were then used to identify barriers and facilitators for effective project management on TTs. In the third round, transcripts were coded based on the five domains of high-performing teams: team management, communication, collaborative problem-solving, affect, and leadership [2]. Any discrepancies were discussed and resolved via consensus.

## Results

### Participants

Fourteen dedicated PMs participated. Like other studies of project management in research teams, most participants were female (79%) [6,15]. While this study was not designed to explore differences in gender, there is evidence in the literature that women are more commonly found in service roles in academic settings [21]. Participants hailed from four CTSA regions, with the majority found in the Midwest (57%). The educational background for participants varied with most completing post-graduate degrees (e.g., Masters, PhD, or MD).

All interviewees were hired to work as dedicated PMs. Some worked in formal PMOs (43%), but the majority did not. Those who were not part of PMOs were either hired to work on specific projects or for specific teams by the PI or were part of smaller programs with less formal infrastructure. Most participants had formal project management training, but only half had formal certification from the Project Management Institute (PMI) [5] (see Table 1). Interestingly, few of the PMs interviewed adopted project management as a primary career choice. In other words, they found their way to project management while pursuing other career paths (e.g., faculty or scientist positions). Once they became aware of their interest and skills in the role, they pursued more formal training in project management. Although the participants had nonlinear career trajectories, they were clearly dedicated to their positions, enthusiastic about their work, and eager to lead TTs to success.

### Identifying barriers and facilitators

**Figure 1.**
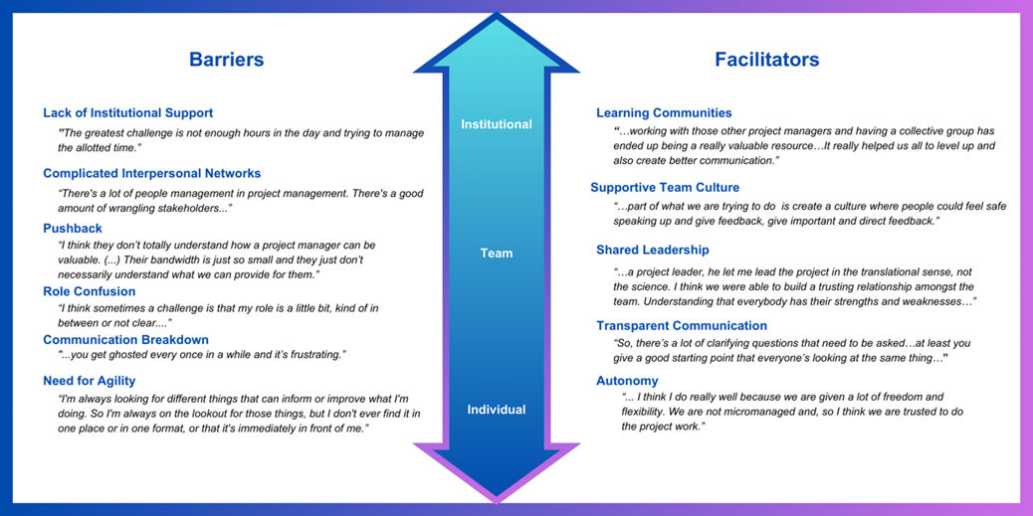
Barriers and facilitators for project management on translational teams. This figure presents the barriers and facilitators for project management on translational teams. The left panel shows the list of barriers to project management identified during the thematic analysis along with representative quotes. The right panel shows the list of facilitators for project management with representative quotes. Both barriers and facilitators are presented as a function of engagement level: institutional, team, and individual.

To effectively implement tailored project management that can accommodate the unique challenges faced by TTs, it is imperative to understand the key determinants that either facilitate or act as a barrier. [20] (see Figure 1).

### Barriers

Six barriers were identified and categorized as institutional, team, or individual level (see Figure 1). PMs commented that there were often not enough resources to support their work. They felt they did not have enough time or bandwidth to adequately meet the needs of their teams. *“I feel like I’m stretched thin across different projects.”* In addition, they did not have easy access to necessary software or tools. *“…I think budget is a barrier as well. [software] licenses are expensive….”* Some PMs indicated that they felt undervalued by their institutions, and this led to problems on their teams in terms of “push-back” from the PIs. The PIs did not understand how the PM could add value, and thought they were there to “check up on the team.”

*“Initially, people thought I was there for oversight. (…) My role is simply to make sure you’re on track and provide you with any service that can help you accomplish this as well as look out for your future.”*

Given the varied ways in which project management is implemented for TTs, it was not surprising to find that role confusion was one of the most mentioned barriers. Either the PI did not understand the PM’s scope of work and/or it was not clear to the PM what they were allowed to do for the team. PMs said things like *“I kind of have this funny position…”* or *“…my role is a little bit, kind of, in between and not clear…”* Clear roles and responsibilities are essential for high-performing teams [22]. Role clarity enhances team members’ levels of satisfaction, performance and innovation [23,24]. Further, when team members identify with their roles and shared vision of the project, they are more motivated and engaged. This alignment between personal and project identity can drive higher levels of commitment and productivity [25]. Without role clarity, confusion in responsibilities causes conflict and results in project delays.

Communication is foundational for team knowledge sharing and project coordination [2]. PMs of TTs must navigate complicated interpersonal networks, dealing with funders, external partners, and team members. Part of this process requires the ability to serve as a knowledge broker essentially “matching” those in the know with those who require the information [26]. Further, some PMs are expected to “serve as proxies” for their busy PIs. Because the leadership of TTs was not a skill for which most PMs received formal training, their work becomes particularly difficult when there are communication breakdowns, especially when they have incomplete information or are completely ignored by TT members or PIs (i.e., “get ghosted.”)

Finally, PMs recognized the need to be versatile. They work with different teams, in different ways, sometimes needing different tools. They often need to find a “blend of soft and hard skills.” Finding the resources they need in terms of training can sometimes be a challenge.

*“I’m always looking for different things that can inform or improve what I’m doing. So, I’m always on the lookout for those things, but I do not ever find it in one place or in one format, or that it’s immediately in front of me.”*

Most of the PMs we interviewed received some kind of formal project management training (13/14), but typically after they were selected for their role. Half of the participants (7/14) were certified through PMI. Most found the content garnered through the certification process useful but found that strict adherence lacked the versatility necessary for TTs. Valenti and colleagues shared similar findings in a 2016 report. For example, they noted that the linear project management approaches commonly found in business fit well with short-term time frames and a clear focus on deliverables. However, academic projects were more likely to have extended time frames and reduced emphasis on financial returns. Thus, they required more nuanced project management approaches [16].

### Facilitators

Five facilitators were identified in this study and categorized as institutional, team, or individual level (see Figure 1). One of the most helpful institutional facilitators was having a community in which PMs could learn and problem solve. These communities could be informal groups or institutionally supported communities of practice.

*“…working with those other project mangers and having a collective group has ended up being a really valuable resource…It really helped us all to level up and also create better communication.”*

PMs worked best in environments where they were accepted as part of the team. They thrived when they were allowed to “share” leadership. “…we really excel when the PIs enable us to help them in any way that we can.” PIs are more likely to do this if they understand the value that an effective PM can bring to their project.

*“…making people understand that I am not trying to tell you what to do. I am not trying to step on toes. You know, we can work together if we do this together, it’s going to be better for everyone. You’re gonna go a lot farther as a team than you are than if just keeping me off to the side. So, it’s really that kind of… getting that buy in. And I think sometimes the best way to get that buy in is people need to actually see the benefit and that takes a while.”*

While PMs should advocate for themselves, institutional support amplifies their efforts and ensures the value of project management is clearly communicated and realized within the organization (e.g., better coordination, higher efficiency, and greater productivity).

Transparent communication was also an important facilitator. Having an environment where information flowed freely, and questions were encouraged was often mentioned. Timely, specific communication is essential for effective team management. This can happen in a variety of ways including email and regular meetings. One person went as far as to suggest using a dashboard tool to create a centralized repository for key project information. Tools like this can make it easier to integrate vast amounts of data and ensure that projects move forward on schedule, within budget.

Finally, it is important to remember that PMs are people with specific interests and skill sets. They found they worked best when given autonomy to choose projects (if possible) and when they were able to do their work unencumbered.

*“Our office and our project leaders I think do really well because we are given a lot of freedom and flexibility. We are not micromanaged and, so I think we are trusted to do the project work.”*

Allowing the opportunity to select projects based on intellectual interests and appropriate skill sets allows for better alignment between the PM and the team. When teams are aligned and there is less conflict, the work can move forward with fewer delays. This is especially true in teams with high levels of self-management [27].

### Managing high-performing teams

Recent articles have articulated the five team-emergent competency domains present when TTs are functioning well. High-performing teams demonstrate effective team management, communication, and collaborative problem-solving, as well as strong affect and leadership [3,22,25]. Enumerated below, effective project management can have an important and impactful influence on TT competency achievement and overall success. PMs may need targeted training to support TT to fully realize these competencies (see Table 3).

**Table 3.** Specific observable behaviors demonstrated by project managers and relevant training for skill enhancement for each of the team-emergent competency domains of high performing teams

Team-emergent competency domain	Components	Specific observable behaviors	Relevant training
**Team management**	Shared visioning Clear roles and responsibilities Effective project management	Develop project plans via Collaboration Planning Use visualizations (e.g., Timelines and Gantt charts) Manage meetings with discipline and strategy	Team science fundamentals Collaboration Planning Basics of project management
**Communication**	Transactive memory systems Shared mental models	Develop organizational charts and central repository systems Hold one-on-one check-ins Facilitate the exchange of expertise among team members	Project management documentation Knowledge brokering
**Collaborative** **problem-solving**	Trans-disciplinarity Collective intelligence Learning/adaptation	Use visualizations to facilitate collaborative approaches to data analysis Conduct regular team debriefs	Facilitation skills Agile practices Team debriefs
**Affect**	Trust Cohesion Psychological safety	Meet one-on-one with each project partner to learn about unique priorities and perspectives Ensure everyone has had a chance to contribute to important discussions Share constructive and compassionate feedback with all team members	Relationship building Active listening, gratitude, and humility
**Leadership**	Goal setting Sense-making Networking Conflict resolution	Facilitate shared goal setting and keep team aligned with shared vision Prioritize needs of team members Connect project partners with needed resources	Leading without authority Servant leadership Needs based conflict resolution

### Team management

The role that PMs play in the management of teams may seem obvious, but the true impact they have on teams often goes unnoticed. Team management is a multifaceted responsibility that extends beyond mere administration; it involves a deep understanding of the project’s overall landscape, anticipating potential challenges, and fostering a collaborative environment which is particularly important for TTs.

*“…my main job is to see the whole picture of the project. (…) not just the whole picture of the science, but the whole picture of the funding and commercialization and the science as well.”*

For a PM, seeing the whole picture is crucial, as it enables them to not only assess the status of the project but also to identify potential risks and opportunities that may arise. This holistic view is essential for making informed decisions and guiding the team towards the successful completion of the project. PMs described Collaboration Planning [12] as one way to help develop the ”bird’s eye view” of their projects.

Collaboration Planning sessions are typically 90 minutes in which a trained facilitator guides an entire TT through a series of questions to help them discuss anticipated challenges and develop strategic plans to overcome them. Discussing roles and responsibilities is a key component of these sessions [12]. As suggested in the previous sections, clear roles and responsibilities for *all* team members are crucial for project success, as they establish a structured framework with clear expectations for each team member. This clarity reduces confusion, prevents overlap in tasks, and enhances accountability, ensuring that everyone is aligned and working towards common goals. When roles are well-defined, team members can focus on their specific duties with confidence, which not only improves efficiency but also fosters a sense of ownership and commitment to the project. Additionally, clear roles facilitate better collaboration, as everyone understands how their contributions fit into the broader objectives, leading to a more cohesive and productive team environment [23,24,28].

### Communication

For TTs, the most important aspects of communication involve integrating knowledge from *all* team members to generate a collective understanding of how to coordinate efforts and ensure alignment of effort. Clarifying roles and responsibilities for team members and establishing a clear transactive memory system is one way that effective PMs can do this. Knowing who does what, who knows what, and how to get things done is integral to a team’s success [29]. PMs acquire and curate the relevant information for each team member and establish the processes through which this information is shared [30].

*“When hiring a project manager. It’s good communication skills. It’s the flexibility and the personality to work with a variety of stakeholders from dean to study coordinator or a project coordinator, you know, early career research scientists and also grad students and postdocs. Because everybody has different needs and understanding…”*

Further developing shared mental models [31], a collective understanding of the TT’s shared goals and expectations, can be challenging especially when working with larger teams. PMs saw success in regular check-ins and meetings. The practice of regular communication between all those involved in the project also allows for proactive problem-solving in the case of “red flags” and issues that may arise rather than solving problems reactively.

One challenge that PMs came across was having a central repository system where all communication and documentation could be managed and stored. One of the most effective ways to manage this is with project management software tools. These tools provide a centralized platform where all project-related information is stored, easily accessible to everyone. This includes timelines, task assignments, progress reports, and communication logs. By maintaining comprehensive documentation, PMs ensure that all team members are informed and aligned with the project’s goals and status. Even if other team members do not fully engage with the repositories themselves, the centralized systems make information easier for the PMs to manage and disseminate more transparently. This transparency reduces the likelihood of misunderstandings and helps keep the project moving forward efficiently [10].

### Collaborative problem-solving

Collaborative problem-solving involves the integration of the unique perspectives of team members with cognitive problem-solving approaches resulting in a novel shared understanding as in the collaborative interpretation of complicated research findings [32]. Effective PMs are skilled facilitators leveraging tools that foster interdisciplinary conversations, like boundary objects. Boundary objects are entities that exist at the intersection of different disciplines or knowledge domains that serve as a shared reference point to enhance communication and problem-solving [33]. Examples include documents, visualizations, or Collaboration Plans [12]. With the “birds-eye view” and well-developed transactive memory system, PMs can help teams more easily explore, analyze, and integrate their collective resources.

PMs can also help TTs learn and adapt to be more versatile. As mentioned by one PM, the COVID-19 pandemic demonstrated just how quickly things can change and the need to rapidly adapt. Agile principles are crucial in today’s fast-paced and ever-changing project environments because they emphasize flexibility, collaboration, and continuous improvement [5]. This approach not only enhances the quality of the outcomes but also fosters a culture of transparency and teamwork, where problems can be identified and addressed early, reducing the risk of costly delays or failures [34].

*“Considering that these projects have a defined time, they really don’t have time to waste sitting around debating whether they should take action. So, I do find that those teams that support Agile are able to show results more quickly and have more time to produce those results.”*

One specific Agile tool that PMs found particularly effective was the team debrief, a structured discussion evaluating the TTs performance in response to a particular event and incorporating these insights into future actions. Engaging the team in regular debriefs provided valuable insights into the project’s dynamics and uncovered hidden issues [35]. Moreover, debriefs enhance team resilience and ability to adapt to disruptive changes [36].

### Affect

High-performing teams have strong cultures. They are cohesive with trusting relationships between individual team members, ultimately resulting in psychological safety [37]. Psychological safety is defined as “…a belief that one will not be punished or humiliated for speaking up with ideas, questions, concerns, or mistakes and that the team is safe for interpersonal risk taking.” [37]. It is a team-emergent phenomenon that must be nurtured by every team member [3,22].

A strong team culture allows each team member the opportunity to fully engage [38]. Participants indicated that they worked hard to get to know each member on the team. In situations where PMs were able to know team members well, they found it easier to facilitate discussions and ensure all voices were heard. Some PMs we interviewed incorporated time after their meetings as a “social hour” where everyone would have the opportunity to talk face-to-face; this “team building” is especially important in larger teams. Gathering information on current preferences and future interests demonstrates the PM’s commitment, building trust between the PM and the team.

By expressing gratitude and humility, skilled PMs can ensure that all team members feel heard [39,40]. This is especially important during team debriefs or feedback sessions. Creating strong and candid feedback loops not only helps in refining processes and strategies but also empowers team members by giving them a voice in the project’s direction. When team members feel heard and valued, their engagement and commitment to the project increase, leading to better overall performance [37].

*“The work that we did was culture change because that’s part of what we’re trying to do, create a culture where people could feel safe speaking up and giving feedback – give important and direct feedback.”*

### Leadership

“Leading and managing a team of strong individuals, trained to work independently, requires a unique skill set to overcome challenges and to successfully achieve goals” [9]. PMs share a part of the leadership in successful TTs. In fact, shared leadership was identified as a facilitator in the previous section. However, as part of the role confusion that was identified as a barrier, it is not always clear to PMs or PIs how to share leadership effectively.

*“It’s a large team with a lot of PIs and a lot of enthusiastic individuals. And everyone wants a say, but no one wants to be the leader. (…) when I saw that dynamic at first I thought, okay, this is a great opportunity for me to step in and be a leader.”*

PMs often need to lead when they are not officially given the power to do so. Leading without authority [41] can be challenging and is not typically something that they are formally trained to do. Thus, PMs need to develop skills like “…navigating difficult conversations, leading without authority, (…) managing the stakeholder piece” if they are going to guide their TTs to success [9].

The role of a PM has been defined as one of a servant leader, embracing a leadership style that prioritizes the needs of others over their own with the goal of improving team well-being [42]. While some assume the practice of servant leadership requires a leadership position, others hold that “anyone who sets out to serve others creates the opportunity to lead those who are served.” [43] The PMs interviewed agreed that the work they did was an act of service and saw themselves as servant leaders. Interestingly, the majority of participants were female which aligns with literature indicating that women are more commonly found in service roles in academic settings [21]. This is particularly relevant as there is increasing evidence that servant leadership, often associated with service roles, positively impacts several team and leadership outcomes including team attitude, team performance, trust in leadership, and perceived leader effectiveness and integrity [42]. Thus, providing training in servant leadership may help enhance PMs’ effectiveness and team impact.

Strong leadership for high-performing teams involves skills in goal setting, sense-making, networking, and conflict resolution [3]. As part of their service to the team, we have already described PMs’ role in shared goal setting and broad networking through the complicated interpersonal networks TTs must navigate. Given their holistic view of the project, they can also help teams make sense of unexpected problems that arise. Some of these problems may include conflicts among broad team partnerships and as such, PMs also need to be skilled in having difficult conversations. These conflicts may arise from differences in power dynamics, suggesting an issue arising from hierarchical leadership structures in which those without positional power are undervalued [3]. This was evident to the PMs we interviewed in the pushback they received and the communication breakdowns they saw (e.g., ghosting). Thus, developing skills in leading without authority and conflict resolution is essential for PMs of TTs.

### Empowering environments for innovative project management

The PMs interviewed for this study were clearly dedicated to their positions and eager to support their teams. Many chose their role as PM while on a more traditional academic pathway because they genuinely enjoyed ushering their teams to success. Based on their unique perspectives, it is not surprising that the descriptions of their work depicted a more expansive view of project management than the more traditional approach. They frequently described situations in which they went well beyond meeting deadlines and managing deliverables. For example, in addition to their deft team management, they described how PMs can help teams develop the skill sets essential for high performance by improving overall communication, fostering psychologically safe and supportive environments, facilitating truly collaborative and innovative problem-solving, all while sharing leadership.

Identifying the key barriers and facilitators for tailored project management is a foundational step towards creating strong environments for PMs and their teams. Based on the interviews with our participants, empowering environments contain three elements.

- **Institutional infrastructure and support** provides training and resources, fostering a culture that understands the value that dedicated project management provides. This value translates into “buy in” from all team members, fostering teams that are more unified and collaborative.
- **Dedicated career pathways** foster flexibility and satisfaction by recognizing project management as a respected professional path and empowering those in these roles with autonomy to influence the projects in their portfolios and share leadership.
- **Learning communities** allow sharing of innovative tools and techniques, learning from experts and collaboratively problem-solving issues as they happen in “real time.” Learning communities, such as communities of practice and centers of excellence, foster essential skill development and promote open information sharing within the complex networks of translational research.

One CTSA that has established an innovative project management program that embraces the more expansive approach is Duke University. Duke University’s clinical and translational enterprise was an early adopter of project management as a resource to support their TTs and has published their project management approaches [6]. Part of Duke’s innovative initiative was to develop a Project Leader Model that defines a team role that includes functions and competencies that go beyond the scope of a more traditional PM. These project leaders are considered an extension of the project PI, bridging scientific and operational domains [6]. Project leaders are supported through a combination of institutional and grant funding and provided training and access to a campus-wide community of practice [44]. Some of the participants in our study served as Project Leaders.

The Project Management Innovation Center of Excellence (PROMICE) recently launched at UW-Madison ICTR leverages the Duke Project Leader experience [6]. PROMICE’s mission is to establish a dedicated career pathway for those supporting project management for translational researchers and translational scientists [45]. The goal is to create a self-sustaining and innovative learning ecosystem to help those in traditional project management roles upskill to better meet the unique needs of TTs. Thus, PROMICE will provide institutional infrastructure and support to amplify the efforts of PMs and ensure the value of their work is clearly communicated and realized. PROMICE’s mission and goals align well with elements of empowering environments identified in this study. A brief description of how PROMICE maps on to each is provided below.

### Institutional infrastructure and support

PROMICE provides training and resources to better enable PMs to meet the needs of their TTs. In addition to developing skills in the basics of project management, PMs benefit from skills in the team emergent competency domains of high-performing teams [22]. For example, to further enhance team management, training in the fundamentals of team science is recommended, and training in knowledge brokering is suggested to enhance communication. Examples of specific observable behaviors for each team-emergent competency domain described by the participants are included in Table 3. The final column in this table also includes participant suggestions for relevant training.

For those on the traditional academic path (e.g., graduate students, postdocs, and early career faculty), team science and leadership competencies like the ones described above and in Table 3 are covered in our TL1 sessions and later reinforced in our complementary team science and training programs like the TT Training Program [46] and K scholar sessions [3]. Training programs like these provide an evidence-based foundation for adaptation for broader audiences like clinical research professionals and dedicated PMs [47]. PROMICE is working to develop similar training specifically targeted for PMs.

### Dedicated career pathway

**Figure 2.**
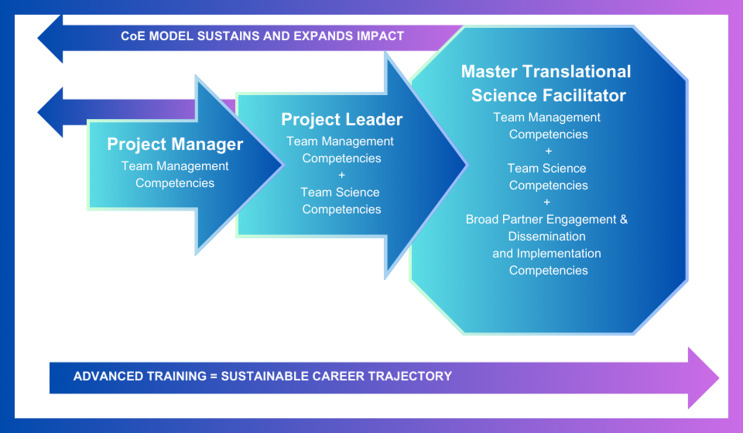
Career pathway for the project management innovation center of excellence (PROMICE). This figure shows how team members in the role of project manager can transition to the role of project leader [6] by acquiring competencies in team science. With further training, they can transition to the role of master translational science facilitator. In addition to demonstrating strong skills in project management and team science competencies, master translational science facilitators are also skilled in dissemination and implementation competencies and broad research partnership engagement competencies. The Center of Excellence (CoE) model utilized by PROMICE allows broader impact using a train the trainer model in which project leaders and master translational science facilitators share what they have learned with those early in the career trajectory [53].

There is much variability in how project management is implemented for TTs across CTSAs, and even within institutions, PMs may hold a variety of official titles (e.g., Research Assistant, PM, Project Leader) [6–8]. This lack of clarity contributes to the role confusion described above and can make it difficult to identify relevant steps for career growth. Creating a dedicated, clearly articulated career pathway is one way to overcome these challenges and streamline efforts towards professional development. PROMICE proposes a three-stage career pathway: 1) Project Manager, 2) Project Leader, and 3) Master Translational Science Facilitator (see Figure 2).

Individuals in the project manger role may be trained in more traditional approaches to project management and are often focused on the deadlines and deliverables of research projects. In addition to becoming skilled in managing translational projects, project leaders need training in team science competencies to promote more effective interdisciplinary collaborations [25]. The PMs within PROMICE will serve in institutional leadership roles as resources for project management, known as master translational science facilitators. To advance to the master translational science facilitator role, participants will gain additional training in broad research partnership engagement and dissemination and implementation. These skills are essential for mature TTs in the translational phase of their intervention to ensure their research findings are effectively communicated, adopted, and sustained, leading to improved outcomes and greater social benefits [48,49].

### Learning communities

PROMICE staff will establish a collaborative learning community modeled after industry Centers of Excellence to help all interested PMs of TTs to be more innovative. In this context, Center of Excellence (CoE) refers to a group that “provides high standards of research, leadership, services or education, and brings innovative mechanisms to promote knowledge and scientific advancements.” [50] CoEs provide specialized subject matter expertise and concentrated resources to cultivate innovation to help organizations stay at the cutting edge of research and technology [50,51]. In contrast to communities of practice, CoEs are strategically created by organizations with dedicated staff members whose primary role is to coach, teach, and mentor [5]. The nascent PROMICE program currently funds two individuals who will provide leadership, experience, and expertise to the broader project management community associated within ICTR. This approach to dissemination is based on the “train the trainer” approach used to share and expand training in mentorship [55,56] (see Figure 2). PROMICE as a CoE will provide training, resources, and infrastructure to help enhance the skills of PMs at all levels.

PROMICE is designed to provide a learning environment to collaborate on translational science projects that contribute to the evidence base of best practices related to TT project management [52]. Going beyond the diffusion of innovation promoted by PMOs [15], CoEs not only disseminate established innovations but provide infrastructure to help develop new innovative approaches. With an emphasis on continuous quality improvement and leveraging translational science, PROMICE staff will conduct research to develop new methods to better lead complex and nuanced team-based projects like those in multisite projects, learning health systems (LHS) [56] and community-engaged research [5].

### Limitations

The PMs interviewed were knowledgeable and provided thorough accounts of their experiences with TTs. However, they form a limited sample of dedicated PMs from CTSAs, and their accounts may not be fully generalizable to all settings. Further, as described above, there is much variability in how project management is implemented for TTs across CTSAs [6–8], and it is possible that our interviews did not capture all models of implementation. While the purposive sampling allowed us to identify dedicated PMs of TTs who were enthusiastic about and fully engaged in their work, it is possible their opinions may not accurately reflect those less engaged or filling the role of PM in a different way. Regardless, the information derived from the interviews serves as a foundation upon which programs can build to empower anyone in the role of PM on TTs.

## Conclusion

High-performing TTs are critical for advancing evidence-based health solutions, but the management of complex teams can be challenging. Further, poor project management may cause more problems for health-related research than methodological issues [4]. Effective project management contributes more to research than the monitoring of deadlines and deliverables, and PMs have the potential to elevate the functioning of teams by improving communication, problem-solving, and culture through shared leadership.

Identifying the key barriers and facilitators for tailored project management is a foundational step towards creating effective environments for PMs and their teams. A greater understanding of TT characteristics that benefit from PM involvement will help to ensure that PMs are integrated and valued team members. The evidence base upon which PROMICE is founded suggests that CoEs can serve to effectively champion project management for TTs by providing institutional support and a collaborative learning community. Through training and collaborative problem-solving, PROMICE as a CoE will empower PMs to foster strong team environments, transparent communication, and enjoy the autonomy they need to enhance TTs’ innovation and impact. Through the development of this dedicated career path, PROMICE recognizes the value that PMs bring to translational science and provides the infrastructure they need to be innovative, leading their teams to success.
